# Endoscopic Nasogallbladder Drainage for Acute Cholecystitis in a Patient With Situs Inversus Totalis: A Case Report

**DOI:** 10.1155/crgm/3485630

**Published:** 2026-10-26

**Authors:** Toji Murabayashi, Mayu Kawabata, Shinya Sugimoto

**Affiliations:** ^1^ Department of Gastroenterology Ise Red Cross Hospital 1-471-2 Funae Ise 516-8512 Mie Japan jrc.or.jp; ^2^ Department of Gastroenterology and Hepatology Mie University Graduate School of Medicine 2-174 Edobashi Tsu 514-8507 Mie Japan mie-u.ac.jp

**Keywords:** acute cholecystitis, case report, endoscopic nasogallbladder drainage, endoscopic transpapillary gallbladder drainage, situs inversus totalis

## Abstract

Situs inversus totalis (SIT) is a rare congenital condition characterized by complete mirror‐image transposition of the thoracic and abdominal organs. Endoscopic transpapillary gallbladder drainage (ETGBD) can be technically challenging in patients with SIT, and, to our knowledge, has not previously been reported in this anatomical setting. We report a case of successful endoscopic nasogallbladder drainage (ENGBD) for acute cholecystitis in a patient with SIT. A 72‐year‐old man with SIT presented with fever and left hypochondrial pain. Computed tomography revealed acute calculous cholecystitis and a common bile duct stone. After removal of the common bile duct stone by endoscopic retrograde cholangiopancreatography, a guidewire was advanced across the obstructed segment of the cystic duct into the gallbladder, and a 6‐Fr ENGBD tube was successfully placed. The patient improved after drainage and subsequently underwent elective laparoscopic cholecystectomy. This case suggests that ETGBD, including ENGBD, may be feasible in patients with SIT when a stable papillary view and guidewire access to the gallbladder can be achieved.

## 1. Introduction

Situs inversus totalis (SIT) is a rare congenital condition characterized by complete mirror‐image transposition of the thoracic and abdominal organs [1]. Endoscopic retrograde cholangiopancreatography (ERCP) in patients with SIT is technically challenging because the endoscopist must adapt to reversed endoscopic and fluoroscopic orientations. Nevertheless, several ERCP‐related procedures, including biliary cannulation, endoscopic sphincterotomy, stone extraction, biliary or pancreatic stent placement, and endoscopic nasobiliary drainage of the bile duct, have been reported in patients with SIT [2–5].

Endoscopic transpapillary gallbladder drainage (ETGBD), including endoscopic nasogallbladder drainage (ENGBD) and endoscopic transpapillary gallbladder stenting, is an established gallbladder drainage option for acute cholecystitis. ETGBD may be considered when percutaneous gallbladder drainage is undesirable or when ERCP is indicated for concomitant biliary disease, such as choledocholithiasis. In such cases, biliary treatment and gallbladder drainage can be performed in the same session [6, 7]. Unlike standard biliary ERCP, ETGBD requires selective cystic duct cannulation and guidewire advancement into the gallbladder, which may be particularly challenging under mirror‐image anatomy. Despite previous reports of ERCP‐related procedures in patients with SIT, ETGBD in this anatomical setting has not been described.

Herein, we report a case of successful ENGBD for acute cholecystitis in a patient with SIT. This case highlights technical considerations for ETGBD under mirror‐image anatomy, including patient positioning, fluoroscopic interpretation, and duodenoscope stabilization.

## 2. Case Presentation

A 72‐year‐old man with known SIT was admitted to our hospital because of fever and left hypochondrial pain. He had no history of hepatobiliary disease or upper abdominal surgery. On admission, his blood pressure was 98/64 mmHg, pulse rate was 73 beats/min, and body temperature was 37.7°C. Physical examination revealed tenderness in the left hypochondrium.

Laboratory tests showed an inflammatory response and hepatobiliary enzyme abnormalities, including a white blood cell count of 9900/μL, C‐reactive protein level of 12.47 mg/dL, aspartate aminotransferase level of 296 U/L, alanine aminotransferase level of 272 U/L, and γ‐glutamyl transpeptidase level of 120 U/L. Serum bilirubin and amylase levels were not elevated. Computed tomography demonstrated SIT, with the liver and gallbladder located on the left side. The gallbladder was distended, with wall thickening and pericholecystic inflammatory changes (Figure 1a), and a common bile duct stone was also identified (Figure 1b). The patient was diagnosed with acute calculous cholecystitis complicated by choledocholithiasis.

**FIGURE 1:**
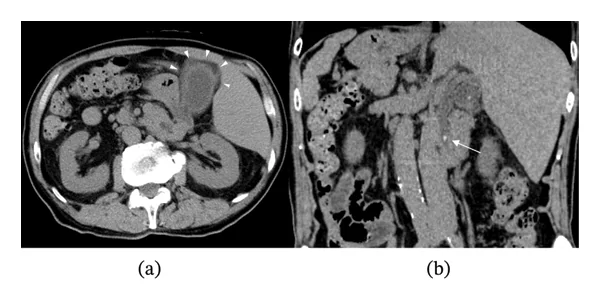
Computed tomography findings on admission. (a) Axial computed tomography image showing situs inversus totalis and a left‐sided inflamed gallbladder. The gallbladder is distended, with wall thickening and surrounding inflammatory changes (arrowheads). (b) Coronal computed tomography image showing a common bile duct stone (arrow) in the setting of mirror‐image abdominal anatomy.

After consultation with the surgical team, the patient was scheduled to undergo endoscopic removal of the common bile duct stone and ETGBD as a bridge to cholecystectomy. ERCP was performed on the day after admission using a standard duodenoscope (TJF‐Q290V; Olympus Medical Systems, Tokyo, Japan). The patient was positioned between the left lateral decubitus and supine positions, with a cushion placed between the patient and the procedure table to maintain the position. After the duodenoscope was advanced to the descending portion of the duodenum, the scope position was adjusted to achieve a stable frontal view of the major papilla. Because maintaining this view required sustained marked leftward torque of the duodenoscope, an additional assistant at the patient’s head held the duodenoscope shaft in the position set by the endoscopist. Any necessary scope adjustments were made by the endoscopist.

Selective biliary cannulation was achieved using a standard cannulation technique with a tapered ERCP catheter (MTW Endoskopie, Wesel, Germany). Cholangiography revealed a common bile duct stone and opacification of the cystic duct up to the obstructed segment, without gallbladder filling (Figure 2a). Endoscopic sphincterotomy followed by endoscopic papillary balloon dilation was performed, and the common bile duct stone was successfully removed using an extraction balloon.

**FIGURE 2:**
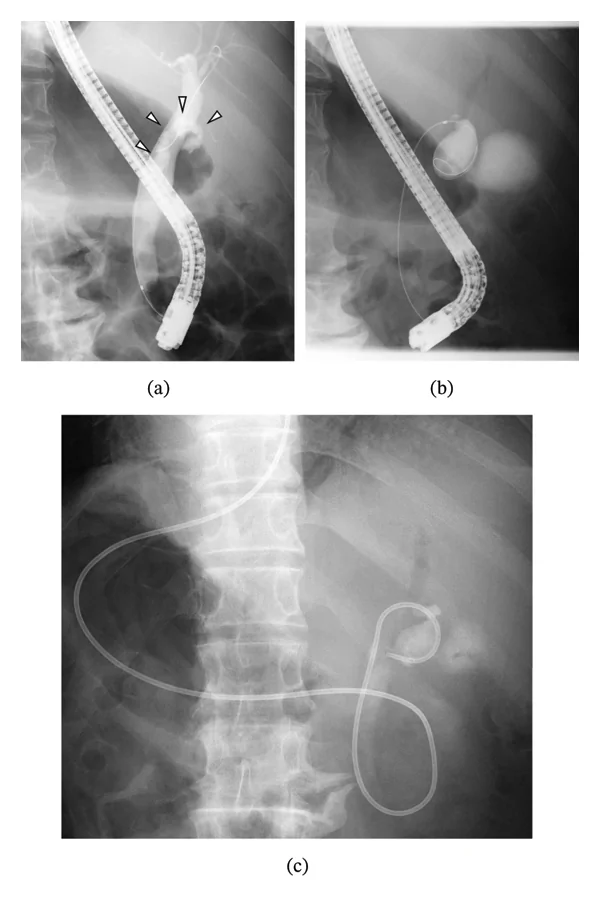
Fluoroscopic findings during endoscopic retrograde cholangiopancreatography and endoscopic nasogallbladder drainage. (a) Cholangiography after selective biliary cannulation showing the bile duct and the partially opacified cystic duct up to the obstructed segment (arrowheads). (b) Fluoroscopic image showing guidewire advancement through the cystic duct and into the gallbladder. (c) Fluoroscopic image showing a 6‐Fr pigtail‐type nasogallbladder drainage tube placed in the gallbladder after duodenoscope withdrawal. Images (a) and (b) were obtained with the patient positioned between the left lateral decubitus and supine positions, whereas image (c) was obtained with the patient in the prone position.

Because common bile duct stone removal was prioritized, ENGBD was attempted after successful stone extraction. The partially opacified cystic duct was used as a fluoroscopic landmark for selective cystic duct cannulation. A 0.025‐inch guidewire (VisiGlide 2; Olympus Medical Systems, Tokyo, Japan) was advanced toward the cystic duct through the same ERCP catheter. The guidewire was successfully passed across the obstructed segment and advanced into the gallbladder (Figure 2b), followed by advancement of the catheter into the gallbladder over the guidewire. A 6‐Fr pigtail‐type nasogallbladder drainage tube (Flexima ENBD Catheter; Boston Scientific, Marlborough, MA, USA) was then placed in the gallbladder over the guidewire (Figure 2c). The gallbladder was angulated at the body, but contrast injected near the gallbladder neck reached the fundus (Figure 2b). We therefore considered deeper placement unnecessary and did not attempt further advancement. The total procedure time, including common bile duct stone extraction and ENGBD, was 56 min.

By the day after ENGBD, the patient’s fever had resolved, abdominal pain had improved, and the white blood cell count had decreased to 5900/μL. The drainage fluid changed from turbid to bile‐colored. Four days after ENGBD, contrast injection through the ENGBD tube showed reduced gallbladder distension and passage of contrast from the gallbladder through the cystic duct into the common bile duct. Given these findings and the patient’s discomfort, the ENGBD tube was removed. No procedure‐related adverse events occurred. Elective laparoscopic cholecystectomy was successfully performed 11 days after ENGBD.

## 3. Discussion

Several ERCP‐related procedures, including biliary cannulation, endoscopic sphincterotomy, stone extraction, biliary or pancreatic stent placement, and endoscopic nasobiliary drainage of the bile duct, have been reported in patients with SIT [2–5]. EUS‐guided gallbladder drainage has also been reported in a patient with SIT [8]; however, this approach does not require transpapillary cystic duct cannulation. To our knowledge, no previous report has described ETGBD, including ENGBD and endoscopic transpapillary gallbladder stenting, in a patient with SIT. Thus, the present case appears to be the first report of ETGBD in this anatomical setting, with ENGBD performed for acute cholecystitis. The main technical implication of this case is that, even in SIT, ETGBD can be completed using standard ERCP accessories once selective cystic duct access and guidewire passage into the gallbladder have been achieved. Therefore, the SIT‐specific challenge lies primarily in maintaining stable papillary orientation and a favorable catheter axis for selective cystic duct cannulation under reversed endoscopic and fluoroscopic orientation.

Patient positioning is another important consideration when performing ERCP in patients with SIT. No standard position has been established, and previous reports have described various approaches, including prone, supine, left lateral, right lateral, and intraprocedural position changes [2, 3]. In the present case, ERCP was performed with the patient positioned between the left lateral decubitus and supine positions. This intermediate position may have balanced three technical considerations. First, because the gallbladder was located on the left side, a left lateral–dominant orientation may have placed the gallbladder in a relatively dependent position, thereby potentially facilitating contrast flow toward the cystic duct and gallbladder. Previous reports on ETGBD have noted that cystic duct direction and cholangiographic visualization of the cystic duct are relevant technical factors for successful ETGBD [7, 9]. Second, a full left lateral decubitus position may make fluoroscopic interpretation of the biliary anatomy more difficult because the biliary tree is projected from a more lateral or oblique angle. Partial rotation toward the supine position may have brought the fluoroscopic image closer to a frontal projection, which may have aided visualization of the bile duct, cystic duct, and guidewire direction. Third, compared to a full prone position, the left lateral–dominant intermediate position may have allowed easier duodenoscope manipulation by the endoscopist. However, these possible advantages were not directly assessed, as different patient positions were not compared in this case. To our knowledge, an intermediate position between the left lateral decubitus and supine positions has not been specifically described for ERCP in patients with SIT. Based on the present experience, this position may represent an additional positioning option when ETGBD is attempted in this anatomical setting.

Scope manipulation and stabilization were also important technical factors in the present case. In SIT, mirror‐image anatomy requires the endoscopist to adapt to reversed endoscopic and fluoroscopic orientations, and substantial torque of the duodenoscope may be necessary to maintain an appropriate papillary view [2, 3]. In the present case, sustained marked leftward torque was required to maintain a stable frontal view of the major papilla. Because maintaining this view was difficult for the endoscopist alone, an additional assistant held the duodenoscope shaft as directed by the endoscopist during the key steps of the procedure. This assistance helped maintain a stable papillary view and catheter axis during biliary cannulation, stone extraction, and subsequent selective cystic duct cannulation. The present case suggests that planned assistance for duodenoscope stabilization may be useful when performing ETGBD or other complex ERCP‐related procedures in patients with SIT. However, these technical observations are based on a single case and should be interpreted with caution.

In conclusion, ETGBD, including ENGBD, may be a feasible option for acute cholecystitis in patients with SIT when a stable papillary view and guidewire passage into the gallbladder can be achieved. Appropriate patient positioning and assisted stabilization of the duodenoscope may contribute to successful ETGBD under mirror‐image anatomy.

## Author Contributions

Conceptualization: Toji Murabayashi. Investigation: Toji Murabayashi and Mayu Kawabata. Supervision: Shinya Sugimoto. Writing–original draft: Toji Murabayashi. Writing–review and editing: Toji Murabayashi, Mayu Kawabata, and Shinya Sugimoto.

## Funding

The authors received no specific funding for this work or for the publication of this article.

## Ethics Statement

Institutional review board approval was not required for this single case report in accordance with local guidelines. Written informed consent was obtained from the patient for publication of this case report and any accompanying images.

## Conflicts of Interest

The authors declare no conflicts of interest.

## Supporting Information

Additional supporting information can be found online in the Supporting Information section.

## Supporting information


**Supporting Information** Completed CARE checklist for this case report.

## Data Availability

The data that support the findings of this study are available on request from the corresponding author. The data are not publicly available due to privacy or ethical restrictions.

## References

[1] Eitler K. , Bibok A. , and Telkes G. , Situs Inversus Totalis: A Clinical Review, International Journal of General Medicine. (2022) 15, 2437–2449, 10.2147/IJGM.S295444. PMC8901252 35264880

[2] Ding B. , Wang J. , Wei X. et al., Efficacy and Safety of ERCP in Patients with Situs Inversus Totalis: Multicenter Case Series and Literature Review, BMC Gastroenterology. (2022) 22, no. 1, 10.1186/s12876-022-02593-3. PMC9714160 36451092

[3] García-Fernández F. J. , Infantes J. M. , Torres Y. , Mendoza F. J. , and Alcazar F. J. , ERCP in Complete Situs Inversus Viscerum Using a ‘Mirror Image’ Technique, Endoscopy. (2010) 42, no. S2, E316–E317, 10.1055/s-0030-1255813. 21113889

[4] Oh C. H. , Successful Endoscopic Retrograde Cholangiopancreatography and Bilateral Stenting in a Patient with Situs Inversus Totalis, Digestive and Liver Disease. (2022) 54, no. 1, 136–137, 10.1016/j.dld.2020.09.019. 33067158

[5] Lee J. M. , Lee H. S. , and Kim C. D. , Infundibulotomy and Endoscopic Retrograde Cholangiopancreatography in Situs Inversus Totalis Combined with Choledochocele, Digestive Endoscopy. (2015) 27, no. 7, 10.1111/den.12514. 26202724

[6] Itoi T. , Kawakami H. , Katanuma A. et al., Endoscopic Nasogallbladder Tube or Stent Placement in Acute Cholecystitis: a Preliminary Prospective Randomized Trial in Japan (With Videos), Gastrointestinal Endoscopy. (2015) 81, no. 1, 111–118, 10.1016/j.gie.2014.09.046. 25527052

[7] Maruta A. , Iwashita T. , Yoshida K. , Shimizu S. , and Shimizu M. , Endoscopic Management of Acute Cholecystitis in high-risk Surgical Patients: a Comprehensive Review Article, DEN Open. (2026) 6, no. 1, 10.1002/deo2.70133. PMC12053924 40330860

[8] Kitagawa S. , Murakoshi N. , and Shiga N. , Endoscopic ultrasound-guided Gallbladder Drainage in Situs Inversus Totalis Using a Loop Formation Technique, Endoscopy. (2026) 58, no. S1, E252–E253, 10.1055/a-2791-4426. PMC12912864 41702574

[9] Sato J. , Nakahara K. , Michikawa Y. et al., The Influence of Pre-procedural Imaging and Cystic Duct Cholangiography on Endoscopic Transpapillary Gallbladder Drainage in Acute Cholecystitis, Diagnostics. (2021) 11, no. 7, 10.3390/diagnostics11071286. PMC8307666 34359369

