# Supplementary material for: Endoscopic Nasogallbladder Drainage for Acute Cholecystitis in a Patient With Situs Inversus Totalis: A Case Report
Source: Case Rep Gastrointest Med. 2026 Oct 26;2026:3485630. doi: 10.1155/crgm/3485630 (PMC13696995; doi:10.1155/crgm/3485630)
Supplement: Supplementary file 1 [file CRGM-2026-3485630-s001.pdf]

CARE Checklist of information to include when writing a case report

CARE case report guidelines

(cc) BY-NC-ND

Topic	Item	Checklist item description	Reported on Line
Title	1	The diagnosis or intervention of primary focus followed by the words “case report”	Lines 1-3
Key Words	2	2 to 5 key words that identify diagnoses or interventions in this case report, including "case report"	Lines 28-30
Abstract (no references)	3a	Introduction: What is unique about this case and what does it add to the scientific literature?	Lines 38 - 42
	3b	Main symptoms and/or important clinical findings	Lines 42-44
	3c	The main diagnoses, therapeutic interventions, and outcomes	Lines 44-48
	3d	Conclusion—What is the main “take-away” lesson(s) from this case?	Lines 48-50
Introduction	4	One or two paragraphs summarizing why this case is unique (**may include references**)	Lines 55-74
Patient Information	5a	De-identified patient specific information.	Lines 79-82
	5b	Primary concerns and symptoms of the patient.	Lines 79-80
	5c	Medical, family, and psycho-social history including relevant genetic information	Lines 79-80
	5d	Relevant past interventions with outcomes	N/A
Clinical Findings	6	Describe significant physical examination (PE) and important clinical findings.	Lines 81-82
Timeline	7	Historical and current information from this episode of care organized as a timeline	Lines 79-125
Diagnostic Assessment	8a	Diagnostic testing (such as PE, laboratory testing, imaging, surveys).	Lines 83-90, 101-105
	8b	Diagnostic challenges (such as access to testing, financial, or cultural)	N/A
	8c	Diagnosis (including other diagnoses considered)	Lines 89-90
	8d	Prognosis (such as staging in oncology) where applicable	N/A
Therapeutic Intervention	9a	Types of therapeutic intervention (such as pharmacologic, surgical, preventive, self-care)	Lines 91-125
	9b	Administration of therapeutic intervention (such as dosage, strength, duration)	Lines 93-118
	9c	Changes in therapeutic intervention (with rationale)	N/A
Follow-up and Outcomes	10a	Clinician and patient-assessed outcomes (if available)	Lines 119-125
	10b	Important follow-up diagnostic and other test results	Lines 119-123
	10c	Intervention adherence and tolerability (How was this assessed?)	Lines 123-124
	10d	Adverse and unanticipated events	Lines 124
Discussion	11a	A scientific discussion of the strengths AND limitations associated with this case report	Lines 129-173
	11b	Discussion of the relevant medical literature **with references**.	Lines 129-161
	11c	The scientific rationale for any conclusions (including assessment of possible causes)	Lines 129-173
	11d	The primary “take-away” lessons of this case report (without references) in a one paragraph conclusion	Lines 174-177
Patient Perspective	12	The patient should share their perspective in one to two paragraphs on the treatment(s) they received	Not obtained
Informed Consent	13	Did the patient give informed consent? Please provide if requested	Yes ☑ No ☐
